# Beyond Analytical Agreement: Integrating Analytical Performance and Algorithm-Assisted Morphological Interpretation in a Comparative Evaluation of the Mindray BC-7800/BC-7900 and Sysmex XN-9100 Hematology Platforms

**DOI:** 10.3390/diagnostics16162642

**Published:** 2026-08-19

**Authors:** Sara Ciullini Mannurita, Domenico Romeo, Alessandro Bonari, Edda Russo, Pamela Nardiello, Valentina Becherucci, Daniela Vitali, Alessandra Fanelli, Francesca Romano

**Affiliations:** 1 General Laboratory, Azienda Ospedaliero-Universitaria Careggi, 50134 Florence, Italydomenico.romeo@unifi.it (D.R.);; 2 Clinical Pathology Laboratory Unit, S. Giuseppe Hospital, Azienda USL Toscana Centro, 50053 Empoli, Italy; 3 Clinical Pathology Laboratory, Santa Maria Annunziata Hospital, 50134 Florence, Italy; valentina.becherucci@uslcentro.toscana.it

**Keywords:** automated hematology analyzers, algorithm-assisted morphology, morphological flagging, complete blood count, leukocyte differential, analyzer comparison, peripheral blood smear, optical microscopy, laboratory validation, diagnostic workflow

## Abstract

**Background/Objectives:** Modern automated hematology analyzers combine quantitative cell counting with proprietary algorithms for automated morphological interpretation. While analytical validation traditionally focuses on measurement agreement, less attention has been paid to the evaluation of algorithm-assisted morphological interpretation. This study compared the analytical performance and automated morphological flagging of the Sysmex XN-9100 and Mindray BC-7800/BC-7900 platforms under routine laboratory conditions. **Methods:** A total of 183 peripheral blood samples collected between October 2025 and February 2026 were analyzed in parallel using both platforms. The study population included healthy individuals and patients with hematological and non-hematological disorders. Analytical agreement for complete blood count (CBC) and leukocyte differential parameters was assessed using Passing–Bablok regression and Bland–Altman analyses. Automated morphological flagging was evaluated in 157 samples using expert optical microscopy as the reference standard. McNemar’s test, Cohen’s kappa coefficient, sensitivity, specificity, positive predictive value (PPV), negative predictive value (NPV), and diagnostic accuracy were calculated. **Results:** Excellent agreement was observed for the principal CBC parameters, including white blood cell count, red blood cell count, hemoglobin, hematocrit, mean corpuscular volume, and platelet count (correlation coefficients: 0.981–1.000), with minimal proportional bias. Greater variability was found for monocytes, eosinophils, and basophils, particularly at low cell concentrations. Although analytical performance was highly comparable, the two platforms showed different morphological flagging profiles. Both analyzers achieved high negative predictive values (>90%), while differences in sensitivity and specificity across individual flag categories reflected distinct algorithm-assisted classification strategies. Agreement with expert microscopy ranged from fair to moderate for most pathological flags. **Conclusions:** The Mindray BC-7800/BC-7900 and Sysmex XN-9100 demonstrated excellent analytical agreement for routine CBC testing. However, comparable analytical performance did not necessarily correspond to identical automated morphological interpretation. These results indicate that evaluation of modern hematology analyzers should include both analytical performance and agreement between automated flags and expert microscopy.

## 1. Introduction

Automated hematology analyzers have progressively evolved from electronic cell counters into advanced analytical platforms capable of combining quantitative blood cell analysis with automated morphological assessment based on fluorescence flow cytometry and multidimensional scattergram interpretation [1,2,3,4]. In addition to providing complete blood count (CBC) parameters and leukocyte differential counts, modern systems incorporate proprietary classification algorithms that support the identification of abnormal cellular populations through automated morphological flags. These developments have transformed automated hematology from a purely analytical discipline into a decision-support tool that assists laboratory professionals in sample prioritization and in selecting specimens requiring microscopic review, while expert morphological examination remains essential for definitive interpretation [5,6,7,8,9]. The analytical performance of modern hematology analyzers has been extensively investigated, demonstrating high precision and reliability for major CBC parameters and leukocyte differential counts. However, comparative studies have also highlighted differences among analytical platforms, particularly regarding leukocyte differential results and automated morphological flagging [5,10,11,12]. These findings indicate that instruments with comparable analytical performance may provide different interpretations of abnormal cellular patterns due to differences in instrument architecture and proprietary classification strategies. Among currently available systems, the Sysmex XN series and the Mindray BC-7000 series represent advanced hematology platforms integrating fluorescence-based technologies and multidimensional signal analysis for automated cellular characterization [5,6,13]. In detail, the Sysmex XN series incorporates dedicated analytical channels for leukocyte characterization and generates morphological flags associated with abnormal lymphoid populations and circulating blasts, whereas the Mindray BC-7000 series applies proprietary fluorescence and scatter-based technologies for cellular discrimination and abnormal population recognition [6,13]. Although both platforms aim to improve automated morphological screening, differences in classification strategies may influence the detection and interpretation of abnormal cellular populations. Current recommendations emphasize that automated morphological flags should always be interpreted together with peripheral blood smear evaluation, particularly in samples suspected of containing blasts, abnormal lymphoid populations, or other clinically significant abnormalities [14,15]. Moreover, previous investigations have demonstrated the clinical usefulness of automated flagging systems; however, most comparative studies have focused primarily on analytical agreement rather than on how different algorithmic approaches influence morphological interpretation and their concordance with expert microscopy [7,8,9,10,11,15]. Despite the increasing integration of algorithm-assisted morphological interpretation into modern hematology analyzers, limited evidence is available regarding the comparative evaluation of different analyzer platforms under routine clinical conditions, particularly when considering both quantitative analytical performance and automated recognition of abnormal cellular patterns.

Therefore, this study aimed to compare the Sysmex XN-9100 and Mindray BC-7800/BC-7900 platforms, focusing on analytical agreement and the performance of automated morphological flagging. We hypothesized that comparable analytical performance between hematology analyzers does not necessarily result in equivalent morphological interpretation because proprietary classification algorithms may influence the recognition of abnormal cellular populations. Accordingly, this study aimed to evaluate analytical agreement of CBC and leukocyte differential parameters and to systematically compare algorithm-dependent morphological interpretation, including automated flags and concordance with expert optical microscopy. This study differs from previous comparisons by combining routine analytical evaluation with an assessment of algorithm-dependent morphological interpretation between two hematology analyzer platforms.

## 2. Materials and Methods

### 2.1. Study Design and Study Population

This was a prospective method-comparison study designed to evaluate the analytical agreement and automated morphological screening performance of the Mindray BC-7800/BC-7900 series (Mindray Bio-Medical Electronics Co., Shenzhen, China) against the Sysmex XN-9100™ system (Sysmex Corporation, Kobe, Japan), which represents the reference hematology platform routinely used at the Central Laboratory of Careggi University Hospital (Florence, Italy).

The aim of the present study was a comparative performance evaluation under routine laboratory conditions rather than a complete analytical validation of instrument performance. Therefore, repeatability, within-run precision, and between-run precision assessments were not included in the study design.

Peripheral blood samples submitted for routine hematological testing between October 2025 and February 2026 were consecutively enrolled. A total of 183 EDTA-anticoagulated whole blood samples were included in the analytical comparison. Samples collected according to routine laboratory procedures were included if they had sufficient volume and were considered suitable for hematological analysis. Samples with clots, insufficient volume, or pre-analytical conditions affecting sample quality were excluded. The study population was intentionally selected to represent the broad spectrum of samples encountered in routine clinical practice and included both normal and pathological specimens.

Specifically, the study cohort comprised 48 samples with normal complete blood count findings, 12 patients with acute leukemia (AL), 16 with myeloid neoplasms (MNs), 24 with lymphoproliferative disorders (LDs), 9 patients undergoing post-bone marrow transplantation monitoring (BMT), and 74 additional samples from patients with solid tumors, infectious diseases, and other medical conditions admitted to cardiology, neurology, orthopedics, and other hospital wards.

Among acute leukemia cases, 11 were acute myeloid leukemia and one was B-cell acute lymphoblastic leukemia. The myeloid neoplasm group included nine myeloproliferative neoplasms, four myelodysplastic syndromes, and three chronic myeloid leukemia cases. Lymphoproliferative disorders included multiple myeloma (*n* = 5), chronic lymphocytic leukemia (*n* = 4), monoclonal B-cell lymphocytosis (*n* = 4), mantle cell lymphoma (*n* = 2), T-cell large granular lymphocytic leukemia (*n* = 3), Hodgkin lymphoma (*n* = 1), and non-Hodgkin lymphoma (*n* = 5). All hematological diagnoses were established according to current WHO diagnostic criteria through integrated clinical, immunophenotypic, histopathological, cytogenetic, and molecular investigations.

The study was conducted in accordance with the ethical principles of the Declaration of Helsinki and was approved by the Area Vasta Centro Ethics Committee (approval No. 13725_bio).

### 2.2. Hematological Analysis

Peripheral blood samples were collected in K2-EDTA tubes (Becton Dickinson, Franklin Lakes, NJ, USA) according to standard pre-analytical procedures and analyzed within six hours of collection.

Each specimen was processed in parallel on both analytical platforms to minimize potential pre-analytical variability. The reference system consisted of the Sysmex XN-9100™, whereas the comparator platform included the Mindray BC-7800 and BC-7900 analyzers.

Instrument settings, including Q-flags and alert thresholds, were maintained at the manufacturers’ recommended default configuration throughout the study. Samples were analyzed using the standard whole-blood analytical mode on each platform (WHOLE BLOOD mode for Sysmex, Whole Blood CDR mode for the BC-7800, and Whole Blood CD + HMC mode for the BC-7900).

The comparison included all routinely certified hematological parameters: white blood cell (WBC) count, red blood cell (RBC) count, hemoglobin (Hb), hematocrit (HCT), mean corpuscular volume (MCV), mean corpuscular hemoglobin (MCH), mean corpuscular hemoglobin concentration (MCHC), red cell distribution width (RDW), platelet count (PLT), mean platelet volume (MPV), neutrophils, lymphocytes, monocytes, eosinophils, basophils (absolute and percentage values), nucleated red blood cells (NRBCs), and reticulocytes (RET).

In addition to conventional CBC parameters, automated morphological alerts generated by the proprietary analytical algorithms of each platform were collected for subsequent comparison with expert optical microscopy.

Manual differential leukocyte counting was performed on 200 cells per peripheral blood smear by a single experienced cytomorphologist using the Nikon H550S optical microscope (Nikon Instruments Inc., Melville, NY, USA), according to CLSI H20-A2 guidelines [12]. The same expert performed all morphological classifications to ensure consistency and minimize inter-observer variability, which represents a recognized limitation of conventional microscopy. Since only one observer was involved, inter-observer agreement was not assessed, and no consensus procedure was required.

The same cytomorphologist also performed the morphological review of digital peripheral blood smear images generated by the DI-60 digital cell imaging analyzer (Sysmex Corporation, Kobe, Japan), which represented the morphological reference standard for the evaluation of automated Q-flags. The use of multiple independent observers could provide additional information regarding observer variability; however, this was beyond the scope of the present study.

### 2.3. Method Comparison

Analytical agreement between the two hematology platforms was evaluated using Passing–Bablok regression analysis for each quantitative parameter. Data distribution was assessed using the Shapiro–Wilk test. Pearson’s correlation coefficient was applied for normally distributed variables, whereas Spearman’s rank correlation coefficient was used for variables not satisfying the normality assumption. Regression slope, intercept, and the corresponding 95% confidence intervals (CIs) were calculated. The overall bias, expressed as a percentage, was evaluated by plotting the values on the axis (method A − method B)/mean (method A − method B) against the mean of the two measurements (Bland–Altman plots). Additional plots showing the mean difference in absolute metric units were generated to better estimate the numerical deviation between method B, considered the reference method (Sysmex XN-9100), and method A (Mindray 7000 series, Shenzhen Mindray Bio-Medical Electronics Co., Ltd., Shenzhen, China).

Statistical analyses and graphical representations were performed using Python (version 3.10) [16] within the Visual Studio Code environment (version 1.110.0) [17].

### 2.4. Evaluation of Automated Morphological Flagging

Automated morphological flagging generated by the two analytical platforms was evaluated against expert peripheral blood smear review, which served as the reference standard.

For each sample, 200 leukocytes were analyzed on peripheral blood smear images using the DI-60 digital cell imaging analyzer. The acquired images were reviewed and reported by the same experienced cytomorphologist who performed the manual differential count.

The evaluator had specific expertise in hematological morphology and had completed dedicated training in hematological morphology, including a final competency assessment.

Morphological evaluation was performed according to the criteria established by the International Society for Laboratory Hematology (ISLH) guidelines.

The hematologist performing the morphological evaluation was not blinded to the analyzer-generated flags as the review was conducted according to the routine laboratory workflow, in which instrument-generated information is available to support morphological assessment and clinical decision-making.

Only Q-flags considered analytically comparable according to the manufacturers’ documentation were included in the comparative analysis.

Agreement between automated flagging and optical microscopy was initially assessed using McNemar’s test to identify systematic differences in paired categorical results.

The degree of agreement beyond chance was subsequently evaluated using Cohen’s kappa coefficient (κ), with interpretation based on conventional categories ranging from poor to good agreement.

Diagnostic performance was calculated for each comparable flag category by determining sensitivity, specificity, positive predictive value (PPV), negative predictive value (NPV), and overall diagnostic accuracy using expert microscopic evaluation as the reference method. Ninety-five percent confidence intervals for PPV and NPV were calculated using the Wilson method.

The same Wilson method was applied to calculate 95% confidence intervals for sensitivity, specificity, and diagnostic accuracy estimates.

A two-sided *p*-value < 0.05 was considered statistically significant.

## 3. Results

### 3.1. Analytical Agreement for Complete Blood Count and Leukocyte Differential Parameters

**Figure 1:**
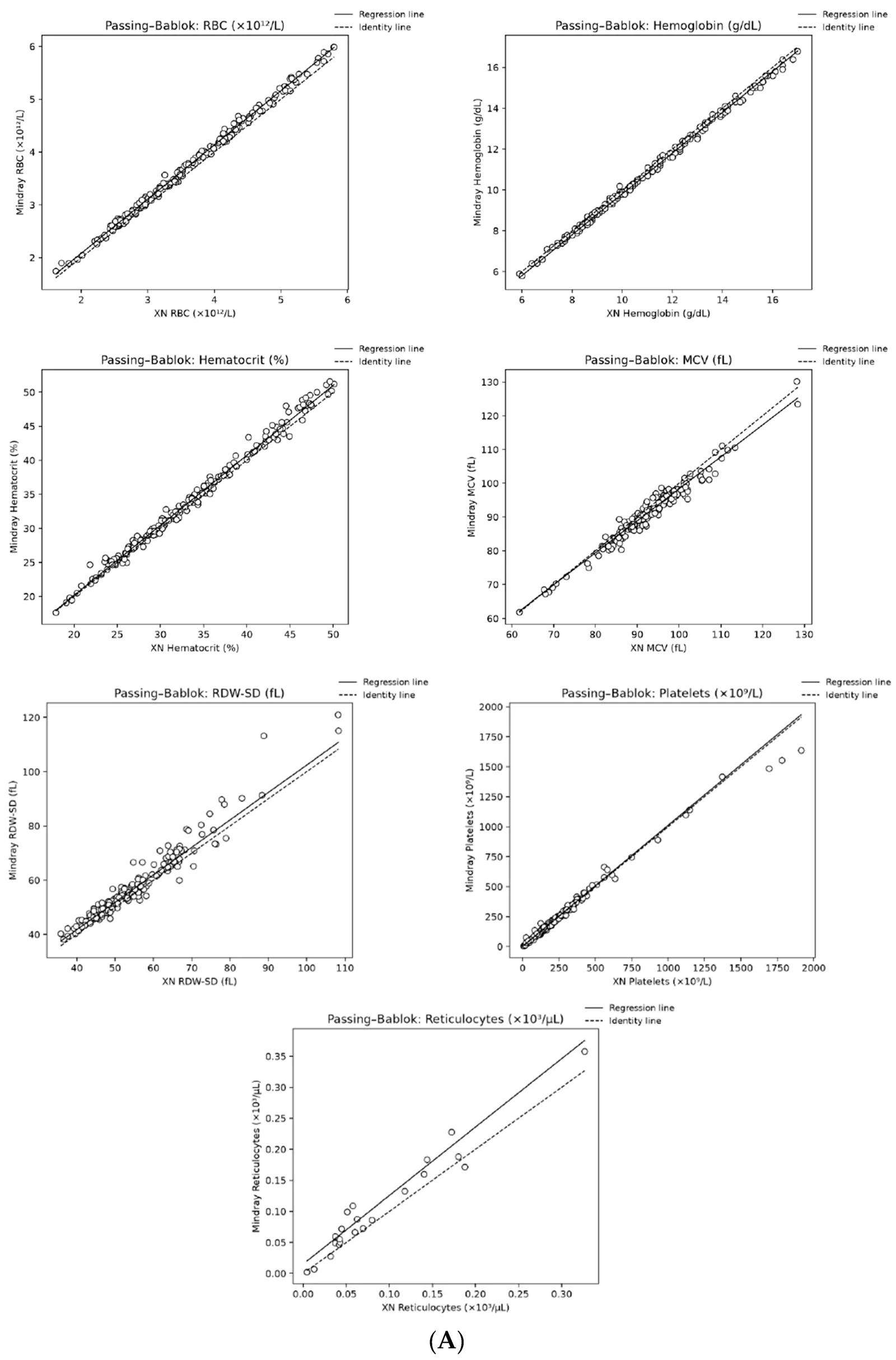

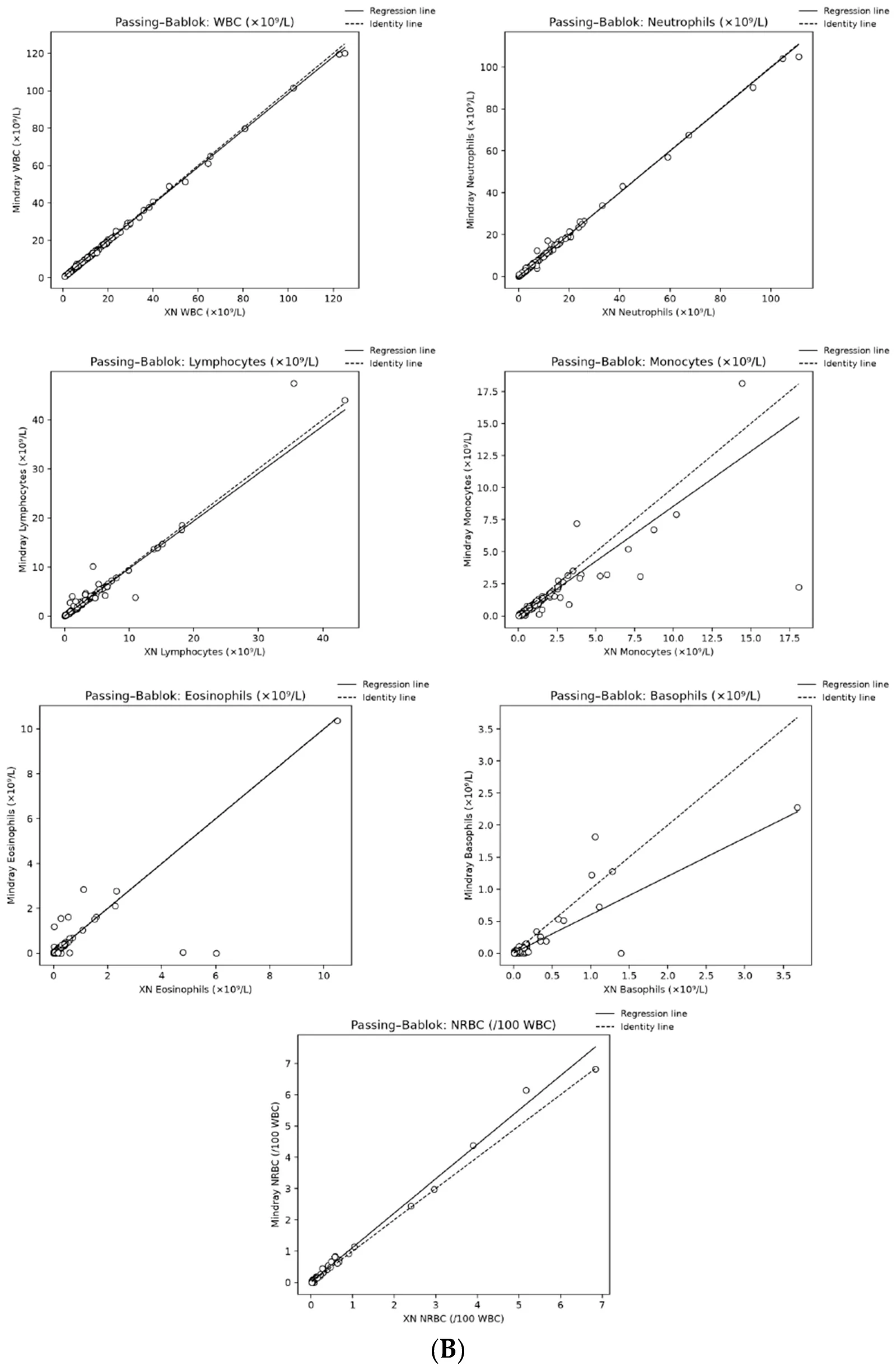

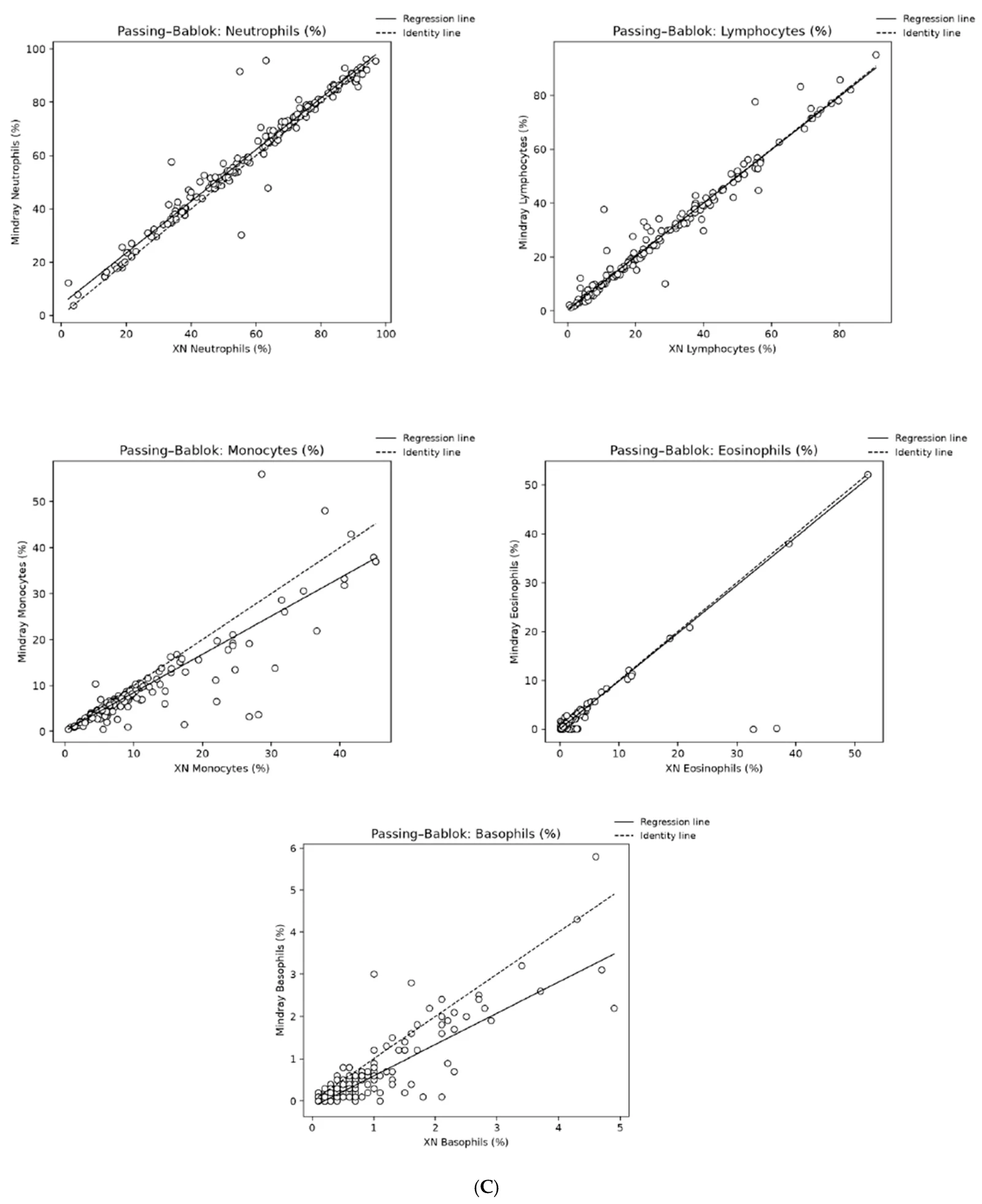
Passing–Bablok regression plots comparing hematological parameters measured by the XN 9100 and Mindray 7000 series analyzers. (**A**) RBCs, Hb, HCT, MCV, RDW-SD, PLT, IGs and RET. (**B**) WBCs, NEUT, LYMPH, MONO, EO, and BASO expressed as absolute counts. (**C**) NEUT%, LYMPH%, MONO%, EO%, and BASO% expressed as percentages.

**Figure 2:**
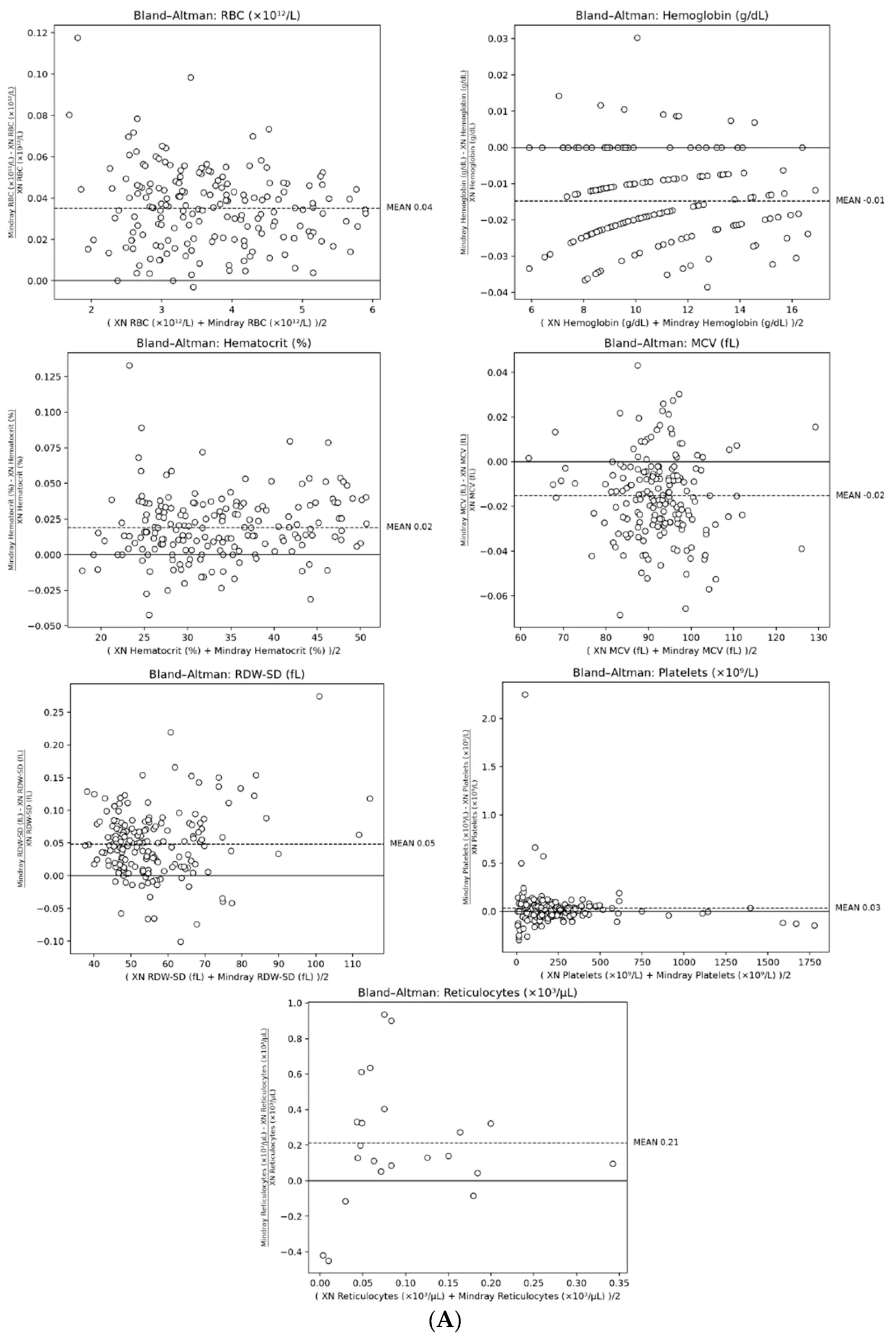

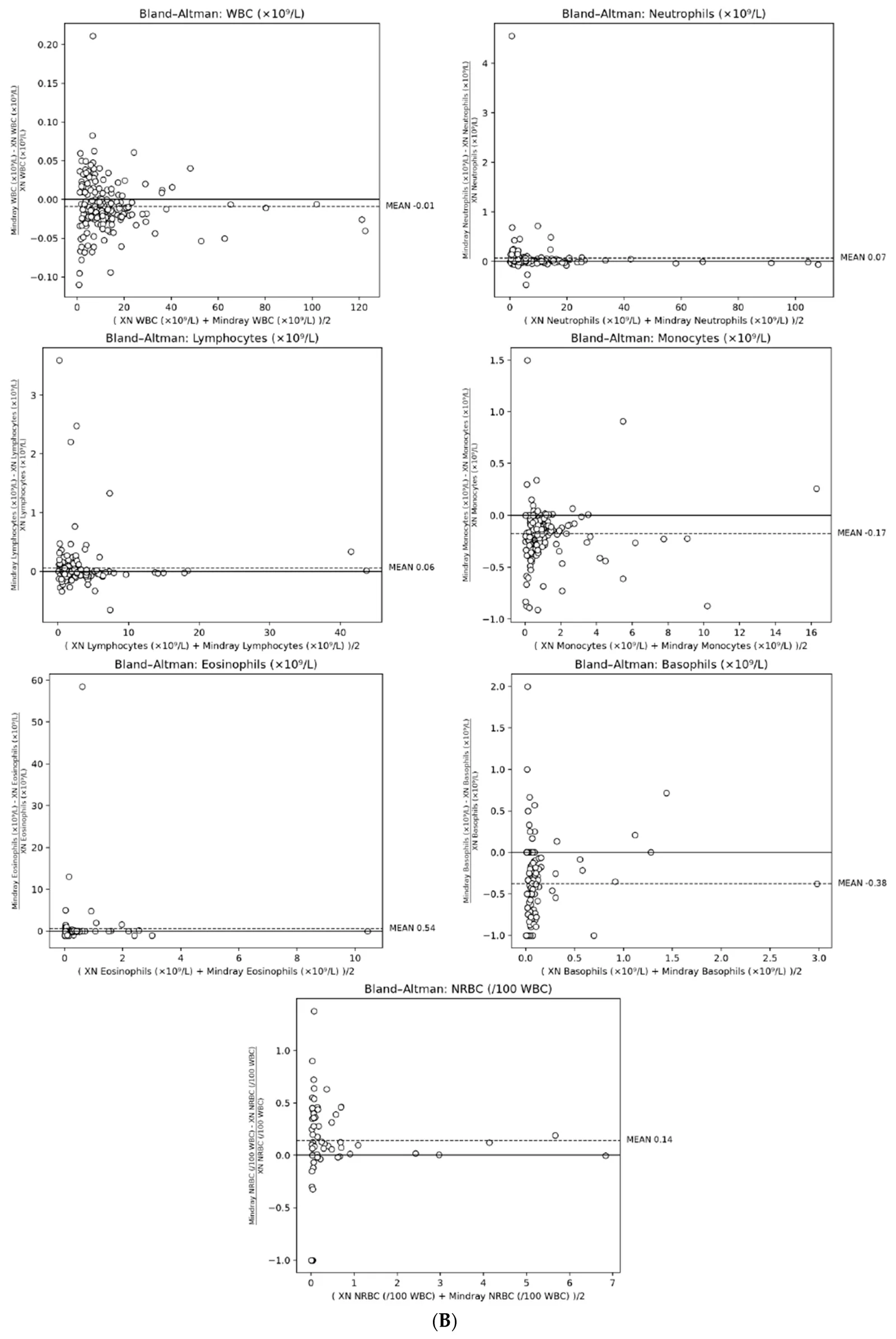

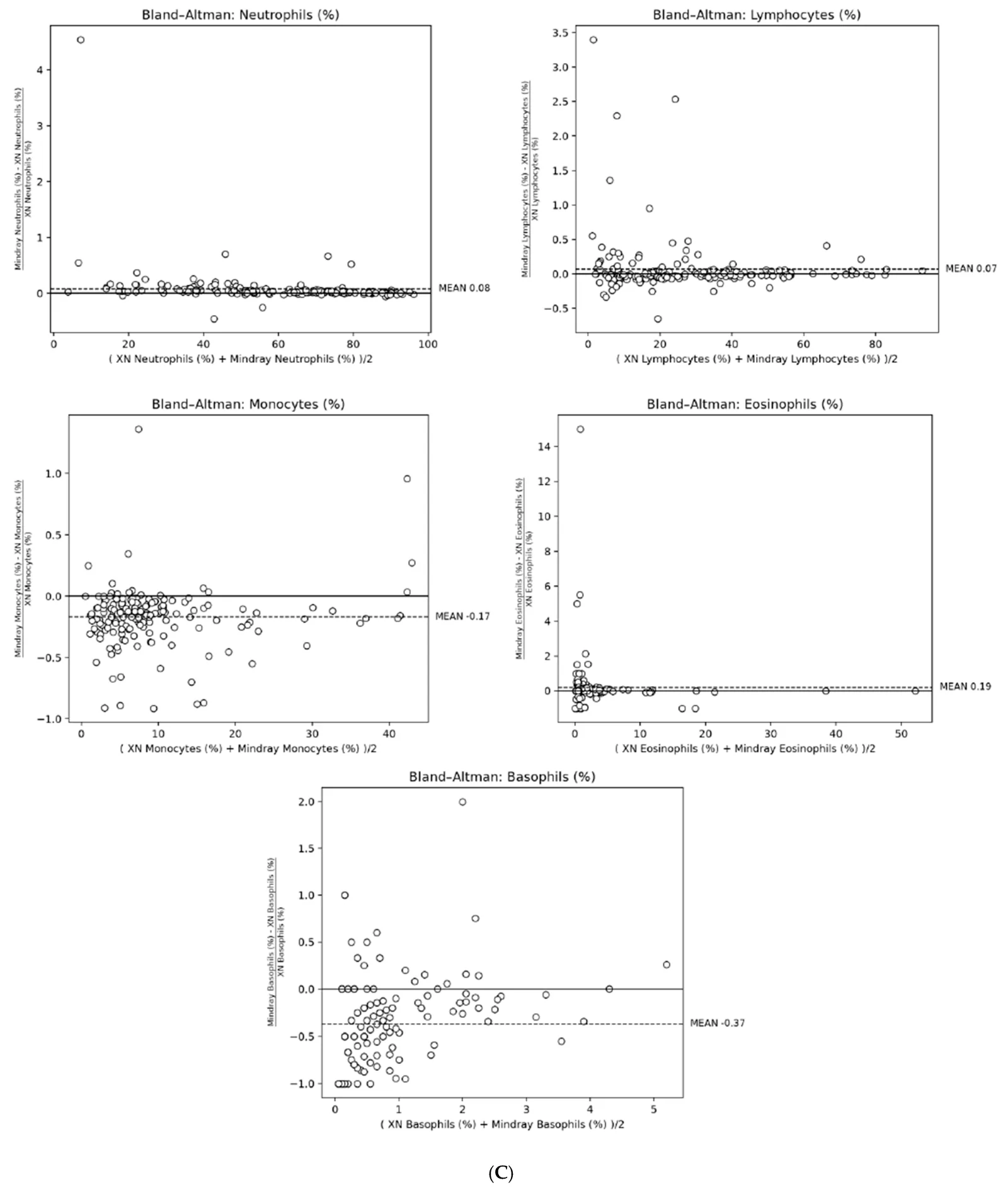
Bland–Altman plots showing the agreement between the XN 9100 and Mindray 7000 series analyzers. (**A**) RBCs, Hb, HCT, MCV, RDW-SD, PLT, IGs, and RET. (**B**) WBCs, NEUT, LYMPH, MONO, EO, and BASO expressed as absolute counts. (**C**) NEUT%, LYMPH%, MONO%, EO%, and BASO% expressed as percentages.

A total of 183 paired peripheral blood samples were included in the analytical comparison, while leukocyte differential counts were available for 176 samples. Overall, the Mindray BC-7800/BC-7900 and Sysmex XN-9100 platforms demonstrated excellent analytical agreement for routine complete blood count (CBC) parameters (Table 1, Figure 1 and Figure 2). According to the distribution of the data assessed by the Shapiro–Wilk test, Pearson’s correlation coefficient was calculated for MCV, MCH, MCHC, and reticulocyte count, whereas Spearman’s rank correlation coefficient was calculated for all other parameters. Agreement between the two analytical systems was further evaluated using Passing–Bablok regression analysis to assess proportional and constant bias, and Bland–Altman analysis to evaluate systematic differences and limits of agreement. Correlation analysis demonstrated excellent agreement for the principal CBC parameters. WBC, RBC, hemoglobin, hematocrit, and platelet counts showed correlation coefficients ranging from 0.993 to 0.999, with regression slopes close to unity (0.95–1.01) and intercepts close to zero, indicating minimal proportional and constant bias between the two analytical systems (Table 1, Figure 1). Bland–Altman analysis further confirmed the high analytical agreement, showing low mean bias and satisfactory limits of agreement, with mean percentage bias below 10% for all major CBC parameters (Table 1, Figure 2).

Among erythrocyte indices, MCV and RDW-SD showed excellent agreement, whereas MCH, MCHC, and MPV demonstrated slightly higher variability without clinically relevant bias. Mean platelet volume (MPV) exhibited the lowest agreement among erythrocyte and platelet indices (r = 0.84), although the regression slope remained close to unity (1.04) and Bland–Altman analysis demonstrated negligible mean bias (Table 1, Figure 1 and Figure 2).

Analysis of the leukocyte differential demonstrated excellent agreement for the major cellular populations. Neutrophils and lymphocytes showed strong agreement (correlation coefficients: 0.994–0.975), regression slopes ranging from 0.97 to 1.01, and minimal mean bias (−6% to +7%). In contrast, greater variability was observed for low-frequency leukocyte populations. Monocytes exhibited the lowest agreement (ρ = 0.96), together with a regression slope of 0.86 and a mean bias of −17%, indicating both proportional bias and reduced analytical agreement. Greater variability was observed for low-frequency leukocyte populations, including eosinophils and basophils, likely reflecting the analytical challenges associated with low cell concentrations. Although nucleated red blood cells and reticulocytes demonstrated good overall agreement between the two analyzers (correlation coefficients of 0.952 and 0.975, respectively), Bland–Altman analysis revealed higher relative bias than that observed for routine CBC parameters (+14% and +21%, respectively), reflecting the greater analytical complexity associated with the enumeration of low-abundance cell populations.

Overall, the combined Passing–Bablok regression and Bland–Altman analyses demonstrated excellent analytical agreement for routine CBC parameters and the major leukocyte populations, whereas greater variability was confined to rare cellular subsets, including monocytes, eosinophils, basophils, NRBCs, and reticulocytes.

### 3.2. Comparative Evaluation of Automated Morphological Flagging

To compare the diagnostic behavior of the two analytical platforms during automated morphological screening, Q-flags generated by the Mindray BC-7800/BC-7900 and Sysmex XN-9100 systems were evaluated against expert optical microscopy, which served as the reference standard. A total of 157 samples were included in this analysis after excluding 19 specimens in which direct comparison was not possible because of differences in manufacturer-specific flag definitions.

For the Sysmex platform, morphological screening was based on the WDF channel with reflex analysis through the WPC channel when pathological alerts were generated. The Mindray platform used the CD channel followed, when appropriate, by the HMC channel for further characterization of suspicious leukocyte populations.

Comparison with optical microscopy using McNemar’s test demonstrated statistically significant disagreement between automated flagging and microscopic evaluation for most morphological categories in both analytical platforms (Table 2). However, some notable exceptions were observed. For the Mindray systems, no statistically significant differences were found for platelet aggregates (*p* = 0.11) and abnormal lymphocytes evaluated by the HMC channel (*p* = 0.26), indicating a distribution of discordant results comparable with that obtained by expert microscopic examination. Similarly, for the Sysmex platform, platelet aggregates (*p* = 0.80) and left shift (*p* = 0.08) did not show significant disagreement with microscopy.

Agreement beyond chance was subsequently assessed using Cohen’s kappa coefficient (Table 3). Overall, concordance ranged from slight to substantial according to the specific morphological category analyzed. The highest agreement for the Mindray platform was observed for abnormal lymphocytes detected by the HMC channel (κ = 0.70), corresponding to substantial agreement with microscopy. Moderate agreement was also obtained for left shift (κ = 0.48) and blast detection using the HMC channel (κ = 0.45), whereas there was fair agreement for abnormal lymphocyte/blast flags, blasts detected by the CD channel, and platelet aggregates. Activated lymphocytes showed only slight agreement (κ = 0.10).

For the Sysmex XN-9100, agreement with microscopy was generally lower, ranging from slight to moderate. The best performance was achieved for blasts detected through the WPC channel (κ = 0.46), while fair agreement was observed for left shift (κ = 0.36), abnormal lymphocytes (κ = 0.30), and abnormal lymphocyte/blast flags (κ = 0.29). Similar to the Mindray platform, activated lymphocytes (κ = 0.18) demonstrated limited concordance with microscopic evaluation.

Overall, both analytical platforms demonstrated variable agreement with expert microscopy depending on the morphological abnormality considered. Nevertheless, the Mindray BC-7800/BC-7900 systems generally achieved higher concordance for selected pathological categories, particularly abnormal lymphocytes, whereas both analyzers showed limited agreement for activated lymphocyte detection, highlighting the intrinsic complexity of automated morphological interpretation.

### 3.3. Diagnostic Performance of Algorithm-Driven Morphological Screening

The diagnostic performance of automated morphological flagging was evaluated using expert optical microscopy as the reference method (Table 4). Although both analytical platforms showed excellent agreement for routine CBC parameters, differences were observed in their morphological flagging profiles. Ninety-five percent confidence intervals for sensitivity, specificity, PPV, NPV, and accuracy were calculated using the Wilson method to account for the uncertainty associated with diagnostic performance estimates. Overall, both systems showed consistently high negative predictive values, generally exceeding 90%, supporting their reliability as screening tools for excluding clinically relevant hematological abnormalities. Conversely, positive predictive values were more variable, reflecting the influence of abnormality prevalence and the limited number of positive cases for some morphological categories. The comparison of diagnostic performance parameters highlighted different algorithm-dependent approaches between the two analyzers. The Sysmex XN-9100 generally showed higher sensitivity for selected morphological alerts. In particular, for abnormal lymphocytes or blasts, the Sysmex WNR flag showed higher sensitivity compared with the corresponding Mindray CD flag (90%, 95% CI 84–94 vs. 71%, 95% CI 63–77). Similarly, for abnormal lymphocytes, Sysmex WPC demonstrated higher sensitivity than Mindray HMC channel (86%, 95% CI 79–90 vs. 64%, 95% CI 56–71). These findings indicate a different sensitivity profile of the two platforms for selected lymphoid abnormalities. In contrast, the Mindray BC-7800/BC-7900 systems tended to show higher specificity for some morphological categories. For abnormal lymphocytes detected by the HMC channel, Mindray showed higher specificity compared with Sysmex WPC (99%, 95% CI 95–99 vs. 76%, 95% CI 68–81). However, specificity differences were less consistent across categories, and overlapping confidence intervals indicate that these findings should be interpreted with caution. For blast detection, the two platforms showed comparable diagnostic performance. The dedicated reflex channels demonstrated similar sensitivity and specificity values with overlapping confidence intervals (Mindray HMC sensitivity: 82%, 95% CI: 75–87; Sysmex WPC sensitivity: 82%, 95% CI: 75–87; specificity: 84%, 95% CI 77–89 vs. 85%, 95% CI 78–89), supporting comparable performance for this clinically relevant abnormality. Both platforms showed a negative predictive value of 98% for blast detection, supporting their ability to reliably exclude samples without blasts and confirming their usefulness as screening tools. For other morphological categories, including activated lymphocytes, platelet aggregates, and left shift, differences between platforms reflected different balances between sensitivity and specificity rather than a consistent advantage of one analyzer. Overall, the two systems showed complementary diagnostic profiles: Sysmex tended to favor sensitivity for selected morphological alerts, whereas Mindray showed higher specificity in some categories. The observed differences should therefore be interpreted as distinct algorithm-dependent classification strategies rather than evidence of superior diagnostic performance of one platform over the other. Both analyzers provided effective support for routine laboratory screening, with their respective characteristics potentially influencing workflow and follow-up decisions in clinical practice.

### 3.4. Overall Analytical and Diagnostic Comparison

Taken together, the results demonstrate that the Mindray BC-7800/BC-7900 and Sysmex XN-9100 platforms achieved highly comparable analytical performance for routine complete blood count parameters and the major leukocyte populations. Greater analytical variability was mainly confined to low-frequency cellular subsets, including monocytes, eosinophils, basophils, nucleated red blood cells, and reticulocytes.

In contrast, the comparative evaluation of automated morphological flagging revealed distinct diagnostic performance profiles between the two analytical systems. While both platforms maintained high negative predictive values and comparable performance for blast detection using their dedicated reflex channels, differences emerged in the balance between sensitivity and specificity across several clinically relevant flag categories. Overall, the Mindray BC-7800/BC-7900 and Sysmex XN-9100 demonstrated different diagnostic profiles during automated morphological screening. The Sysmex XN-9100 generally showed higher sensitivity for selected pathological alerts, whereas the Mindray platform tended to provide higher specificity for some morphological categories. However, diagnostic accuracy values showed variable patterns across flag categories, with overlapping confidence intervals in several comparisons, indicating no overall statistically demonstrated superiority of one platform over the other.

These findings indicate that, despite excellent analytical agreement for conventional hematological measurements, the two analytical platforms generate different automated morphological screening outputs, reflecting differences in their embedded flagging algorithms and diagnostic strategies.

## 4. Discussion

The comparison of analytical performance showed excellent agreement between the Mindray BC-7800/BC-7900 and Sysmex XN-9100 platforms for routine CBC parameters and the main leukocyte populations. As expected, greater variability was observed for less frequent cell populations, particularly monocytes and eosinophils, in agreement with previous studies reporting higher variability for rare leukocyte subsets among automated hematology analyzers [5,10,13]. In our study, monocytes showed the lowest agreement among leukocyte populations (slope: 0.86; mean bias: −17%), suggesting that differences in cellular discrimination may have a greater impact on low-abundance populations. The comparison of automated morphological flagging showed different diagnostic profiles between the two systems. The Sysmex XN-9100 generally showed higher sensitivity for selected alerts, including abnormal lymphocytes or blasts detected by the WNR channel and abnormal lymphocytes detected by the WPC channel, whereas Mindray showed higher specificity in some categories, particularly abnormal lymphocytes evaluated through the HMC channel. These findings indicate different approaches to morphological classification, with different balances between sensitivity and specificity, rather than a clear advantage of one platform over the other. The two analyzers showed comparable performance for blast detection using their dedicated reflex channels; however, the moderate agreement with microscopy confirms that automated flags are intended to support, rather than replace, expert morphological evaluation [6,7,8,9,13,14,15]. Overall, these results indicate that differences between advanced hematology analyzers are not limited to numerical measurements but also involve the interpretation of cellular abnormalities. Automated morphological flagging may improve sample prioritization and laboratory workflow, but microscopic review remains essential for definitive assessment of abnormal findings. Future method comparison studies should therefore consider both conventional analytical performance and the ability of analyzer-specific algorithms to identify morphological abnormalities, including their agreement with expert microscopy and their impact on routine laboratory practice.

## 5. Conclusions

The Mindray BC-7800/BC-7900 and Sysmex XN-9100 platforms demonstrated excellent analytical agreement for routine complete blood count parameters. However, differences were observed in automated morphological flagging, with Sysmex showing higher sensitivity for selected alerts and Mindray showing higher specificity in some categories, resulting in different diagnostic profiles rather than overall superiority of one system. Both analyzers showed high negative predictive values, supporting their reliability as screening tools for excluding clinically relevant abnormalities, while expert microscopy remained essential for definitive morphological assessment. These findings indicate that evaluation of advanced hematology analyzers should consider not only analytical performance but also the agreement between automated morphological flagging and expert microscopic findings.

## Figures and Tables

**Table 1:** Method comparison between the Mindray BC-7800/BC-7900 and Sysmex XN-9100 hematology analyzers.

Parameter	Range XN	Range Mindray	Analytical Range (XN)	Shapiro (*p*-Value)	Pearson (*r*)	Spearman (ρ)	Slope (95% CI)	Intercept (95% CI)	Bias %
WBCs (×10^9^/L)	0.82–125.14	0.73–120.07	0.73–125.14	<0.05	1.0	0.999 *	0.98 (0.98–0.99)	0.08 (−0.13–0.49)	−0.01
RBCs (×10^12^/L)	1.62–5.8	1.75–5.99	1.62–5.99	<0.05	0.998	0.998 *	1.03 (1.01–1.03)	0.03 (−0.06–0.12)	0.04
Hemoglobin (g/dL)	5.9–17.0	5.8–16.8	5.8–17.0	<0.05	0.999	0.999 *	1.0 (0.98–1.0)	−0.2 (−0.25–0.12)	−0.01
Hematocrit (%)	17.9–50.1	17.7–51.6	17.7–51.6	<0.05	0.996	0.996 *	1.03 (1.01–1.04)	−0.39 (−1.29–0.33)	0.02
MCV (fL)	61.8–128.4	61.9–130.2	61.8–130.2	0.39	0.981 *	0.968	0.95 (0.92–0.98)	3.6 (0.51–6.85)	−0.02
MCH (pg)	18.9–44.4	18.1–41.0	18.1–44.4	0.35	0.985 *	0.97	0.88 (0.85–0.9)	2.14 (1.44–3.04)	−0.05
MCHC (g/dL)	28.6–37.7	27.6–34.2	27.6–37.7	0.06	0.866 *	0.864	0.67 (0.59–0.74)	9.83 (7.57–12.49)	−0.03
RDW-SD (fL)	35.8–108.3	38.3–121.0	35.8–121.0	<0.05	0.97	0.965 *	1.01 (0.96–1.06)	1.33 (−1.29–4.02)	0.05
Platelets (×10^9^/L)	6.0–1914.0	6.0–1638.0	6.0–1914.0	<0.05	0.994	0.993 *	1.01 (0.99–1.03)	3.51 (−4.0–17.07)	0.03
MPV (fL)	8.3–13.9	7.4–14.2	7.4–14.2	<0.05	0.84	0.853 *	1.04 (0.95–1.16)	−0.22 (−1.56–0.74)	0
Neutrophils (×10^9^/L)	0.04–111.13	0.04–104.9	0.04–111.13	<0.05	0.998	0.994 *	1.0 (0.99–1.01)	0.05 (−0.32–0.31)	0.07
Lymphocytes (×10^9^/L)	0.03–43.4	0.03–47.44	0.03–47.44	<0.05	0.977	0.975 *	0.97 (0.95–0.98)	0.02 (−0.06–0.17)	0.06
Monocytes (×10^9^/L)	0.03–18.1	0.01–18.14	0.01–18.14	<0.05	0.783	0.96 *	0.86 (0.81–0.89)	−0.03 (−0.07–0.03)	−0.17
Eosinophils (×10^9^/L)	0.01–10.5	0.0–10.36	0.0–10.5	<0.05	0.789	0.739 *	1.0 (0.95–1.0)	−0.01 (−0.06–0.01)	0.54
Basophils (×10^9^/L)	0.01–3.68	0.0–2.28	0.0–3.68	<0.05	0.877	0.651 *	0.6 (0.5–0.67)	0.0 (−0.01–0.01)	−0.38
Neutrophils (%)	2.2–96.8	3.8–96.2	2.2–96.8	<0.05	0.975	0.968 *	0.97 (0.95–0.98)	4.02 (1.03–7.4)	0.08
Lymphocytes (%)	0.5–90.8	1.4–95.1	0.5–95.1	<0.05	0.98	0.978 *	0.99 (0.98–1.0)	0.46 (−1.0–3.97)	0.07
Monocytes (%)	0.5–45.2	0.5–56.0	0.5–56.0	<0.05	0.871	0.872 *	0.83 (0.79–0.87)	0.15 (−0.45–0.56)	−0.17
Eosinophils (%)	0.1–52.2	0.0–52.1	0.0–52.2	<0.05	0.812	0.761 *	0.98 (0.93–1.0)	0.03 (−0.33–0.26)	0.19
Basophils (%)	0.1–4.9	0.0–5.8	0.0–5.8	<0.05	0.855	0.779 *	0.74 (0.63–0.83)	−0.14 (−0.24–−0.05)	−0.37
NRBCs (/100 WBCs)	0.02–6.84	0.0–6.82	0.0–6.84	<0.05	0.996	0.952 *	1.1 (1.02–1.19)	0.02 (−0.01–0.04)	0.14
Reticulocytes (×10^3^/μL)	0.0–0.33	0.0–0.36	0.0–0.36	0.39	0.975 *	0.945	1.1 (0.91–1.32)	0.02 (−0.01–0.04)	0.21

* Recommended correlation coefficient based on Shapiro–Wilk test *p*-value (Pearson’s *r* for *p* > 0.05, Spearman’s ρ for *p* ≤ 0.05).

**Table 2:** Comparison between instrument-generated morphological flags and optical microscopy using McNemar’s test.

Platform	Morphological Flag	McNemar χ^2^	*p*-Value	Interpretation
**Mindray BC-7800/BC-7900**	Abnormal lymphocytes or blasts (CD)	12.10	<0.001 ***	Significant difference
	Blasts (CD)	24.50	<0.001 ***	Significant difference
	Blasts (HMC)	14.44	<0.001 ***	Significant difference
	Activated lymphocytes	12.45	<0.001 ***	Significant difference
	Platelet aggregates	2.57	0.11	Not significant
	Left shift	6.26	0.01 *	Significant difference
	Abnormal lymphocytes (HMC)	1.29	0.26	Not significant
**Sysmex XN-9100**	Abnormal lymphocytes or blasts (WNR)	45.63	<0.001 ***	Significant difference
	Blasts (WPC)	13.50	<0.001 ***	Significant difference
	Activated lymphocytes	37.10	<0.001 ***	Significant difference
	Platelet aggregates	0.07	0.80	Not significant
	Left shift	3.13	0.08	Not significant
	Abnormal lymphocytes (WPC)	29.40	<0.001 ***	Significant difference

* *p* < 0.05; *** *p* < 0.001.

**Table 3:** Agreement between automated morphological flags and optical microscopy as assessed by Cohen’s kappa coefficient.

Morphological Flag	Mindray BC-7800/7900 κ	Interpretation	Sysmex XN-9100 κ	Interpretation
Abnormal lymphocytes or blasts	0.37	Fair	0.29	Fair
Blasts	0.39 (CD)/0.45 (HMC)	Fair/Moderate	0.46 (WPC)	Moderate
Activated lymphocytes	0.10	Slight	0.18	Slight
Platelet aggregates	0.37	Fair	0.16	Slight
Left shift	0.48	Moderate	0.36	Fair
Abnormal lymphocytes	0.70 (HMC)	Substantial	0.30 (WPC)	Fair

**Table 4:** Diagnostic performance of algorithm-driven morphological flagging compared with expert optical microscopy.

Morphological Flag	Platform	Sensitivity		Specificity		PPV		NPV		Accuracy	
		%	95% CI	%	95% CI	%	95% CI	%	95% CI	%	95% CI
**Abnormal** **lymphocytes or blasts**	Mindray (CD)	71	(63–77)	**75**	(68–81)	42	(34–49)	**91**	(85–94)	75	(67–80)
	Sysmex (WNR)	**90**	(84–94)	57	(49–64)	**34**	(27–41)	**96**	(91–98)	64	(55–70)
**Blasts**	Mindray (CD)	88	(82–92)	**79**	(71–84)	33	(26–41)	**98**	(94–99)	80	(72–85)
	Mindray (HMC)	82	(75–87)	**84**	(77–89)	39	(31–46)	**98**	(93–99)	**84**	(77–88)
	Sysmex (WPC)	**82**	(75–87)	**85**	(78–89)	40	(32–47)	**98**	**(93–99)**	**85**	(78–89)
**Activated lymphocytes**	Mindray	38	(30–45)	**84**	(77–88)	11	(7–16)	**96**	**(91–98)**	**82**	(74–86)
	Sysmex	**88**	**(81–91)**	73	(65–79)	15	(10–21)	**99**	**(96–99)**	74	(66–80)
**Platelet aggregates**	Mindray	56	(47–63)	**93**	**(88–96)**	33	(26–41)	**97**	**(93–98)**	**91**	**(85–94)**
	Sysmex	22	(16–29)	**95**	**(89–97)**	20	(14–26)	**95**	**(90–97)**	**90**	**(84–94)**
**Left shift**	Mindray	73	(65–79)	**85**	(78–89)	49	(41–56)	**94**	**(89–96)**	**83**	(76–87)
	Sysmex	58	(49–65)	**84**	(77–88)	42	(34–49)	**91**	**(85–94)**	**80**	(72–85)
**Abnormal lymphocytes**	Mindray (HMC)	64	(56–71)	**99**	**(95–99)**	**82**	(75–87)	**97**	**(92–98)**	**96**	**(91–97)**
	Sysmex (WPC)	**86**	(79–90)	76	(68–81)	26	(19–32)	**98**	**(94–99)**	76	(69–82)

Bold values indicate the highest point estimate for each diagnostic performance metric among the compared platforms.

## Data Availability

The data presented in this study are available on request from the corresponding authors.

## Abbreviations

The following abbreviations are used in this manuscript:

AL	Acute Leukemia
BASO	Basophils
BMT	Bone Marrow Transplantation
CBC	Complete Blood Count
CI	Confidence Interval
DIFF	Leukocyte Differential Count
EDTA	Ethylene Diamine Tetraacetic Acid Dipotassium
EO	Eosinophils
ESR	Erythrocyte Sedimentation Rate
Hb	Hemoglobin
HCT	Hematocrit
HMCs	Hematological Malignant Cells
IGs	Immature Granulocytes
κ	Cohen’s Kappa Coefficient
LDs	Lymphoproliferative Disorders
LYMPH	Lymphocytes
MCH	Mean Corpuscular Hemoglobin
MCHC	Mean Corpuscular Hemoglobin Concentration
MCV	Mean Corpuscular Volume
MNs	Myeloid Neoplasms
MONO	Monocytes
MPV	Mean Platelet Volume
NEUT	Neutrophils
NPV	Negative Predictive Value
NRBCs	Nucleated Red Blood Cells
PLT	Platelets
PLT-F	Fluorescent Platelet Channel
PLT-O	Optical Platelet Count
PPV	Positive Predictive Value
Q-flag	Qualitative Instrument Flag
RBCs	Red Blood Cells
RDW	Red Cell Distribution Width
RET	Reticulocytes
WBCs	White Blood Cells
WDF	White Cell Differential Channel
WNR	White Nuclear Red Channel
WPC	White Progenitor Cell Channel
